# Editorial Comment: Erectile function after partial penectomy for penile cancer

**DOI:** 10.1590/S1677-5538.IBJU.2019.0119.1

**Published:** 2021-03-25

**Authors:** Rodrigo Barros de Castro

**Affiliations:** 1 Universidade Federal Fluminense Hospital Universitário Antônio Pedro Serviço de Urologia NiteróiRJ Brasil Serviço de Urologia, Hospital Universitário Antônio Pedro - Universidade Federal Fluminense - UFF, Niterói, RJ, Brasil.

## COMMENT

Penile cancer is rare in industrialized countries, and the incidence is higher in regions of Africa, Asia and South America (1, 2). Partial penectomy is generally indicated for invasive distal tumor and has a great impact on sexuality, body image and mental health (3). However, there are few studies evaluating the impact of partial penectomy on erectile function of penile cancer patients.

In this interesting paper, the authors evaluate the erectile function of patients who underwent partial penectomy at two centers and identify factors associated with penile functional status. Satisfactory baseline sexual intercourse was obtained during interview prior to surgery and erectile function was assessed according to the International Index of Erectile Function (IIEF-5) after the procedure. Although approximately 62% of the patients had erectile dysfunction (ED) after partial penectomy, the degree of ED was mild in 9 (11.2%) cases, mild to moderate in 17 (21%), moderate in 9 (11.2%), and severe in 15 (18.3%) cases. Interestingly, risk factors for ED such as obesity, hypertension, dyslipidemia and smoking between patients with and without ED was not significantly different. However, smaller penile shaft length, clinically positive lymph node and older age significantly increased the incidence of ED (4).

Male sexuality is a complex phenomenon comprising emotional, physical, and relational aspects and can be deeply affected by cancer treatment (5). The ED etiology may be multifactorial, and psychological aspects can play an important role in these cases. Feelings of shame due to the small penis size and the absence of the glans are some reasons for not resuming sexual intercourse by patients after undergoing partial penectomy (6). Depression, social anxiety and sexual performance anxiety are very common in these patients because of malignant disease and body image changes. According to a recent publication, sexual performance anxiety can affect 9-25% of men and contributes to psychogenic ED (7).

Partial penectomy provides excellent local control of the disease. However, it can psychologically affect patients and impair sexual function, especially erectile function. Therefore, patients should be advised of this possibility.

The present study has some limitations, such as failure to measure psychological problems like depression and anxiety, which are risk factors for ED. However, the authors present the second largest study regarding erectile function in patients who underwent partial penectomy due to penile cancer. The authors deserve congratulations for the important report in this paper.
